# *In Vitro* and *Ex Vivo* Evaluations on Transdermal Delivery of the HIV Inhibitor IQP-0410

**DOI:** 10.1371/journal.pone.0075306

**Published:** 2013-09-18

**Authors:** Anthony S. Ham, William Lustig, Lu Yang, Ashlee Boczar, Karen W. Buckheit, Robert W. Buckheit Jr

**Affiliations:** ImQuest BioSciences Inc., Frederick, Maryland, United States of America; University of California, San Francisco, United States of America

## Abstract

The aim of this study was to investigate the physicochemical and *in vitro/ex vivo* characteristics of the pyrmidinedione IQP-0410 formulated into transdermal films. IQP-0410 is a potent therapeutic anti-HIV nonnucleoside reverse transcriptase inhibitor that would be subjected to extensive first pass metabolism, through conventional oral administration. Therefore, IQP-0410 was formulated into ethyl cellulose/HPMC-based transdermal films via solvent casting. *In mano* evaluations were performed to evaluate gross physical characteristics. *In vitro* release studies were performed in both Franz cells and USP-4 dissolution vessels. *Ex vivo* release and permeability assays were performed on human epidermal tissue models, and the permeated IQP-0410 was collected for *in vitro* HIV-1 efficacy assays in CEM-SS cells and PBMCs. Film formulation D3 resulted in pliable, strong transdermal films that were loaded with 2% (w/w) IQP-0410. Composed of 60% (w/w) ethyl cellulose and 20% (w/w) HPMC, the films contained < 1.2% (w/w) of water and were hygroscopic resulting in significant swelling under humid conditions. The water permeable nature of the film resulted in complete *in vitro* dissolution and drug release in 26 hours. When applied to *ex vivo* epidermal tissues, the films were non-toxic to the tissue and also were non-toxic to HIV target cells used in the *in vitro* efficacy assays. Over a 3 day application, the films delivered IQP-0410 through the skin tissue at a zero-order rate of 0.94 ± 0.06 µg/cm^2^/hr with 134 ± 14.7 µM collected in the basal media. The delivered IQP-0410 resulted in *in vitro* EC_50_ values against HIV-1 of 2.56 ± 0.40 nM (CEM-SS) and 0.58 ± 0.03 nM (PBMC). The film formulation demonstrated no significant deviation from target values when packaged in foil pouches under standard and accelerated environmental conditions. It was concluded that the transdermal film formulation was a potentially viable method of administering IQP-0410 that warrants further development.

## Introduction

With over 25 million deaths attributed to AIDS since the first cases in 1981, 33 million individuals worldwide living with HIV, and over 2.5 million new infections yearly, HIV/AIDS continues to be a global emergency [1]. To combat this epidemic, combinations of nucleoside, nucleotide and nonnucleoside reverse transcriptase inhibitors and protease inhibitors have been effectively used in highly active anti-retroviral therapies (HAART) to significantly reduce HIV virus load in infected individuals for prolonged periods of time. The utilization of HAART has dramatically changed the therapeutic landscape of HIV treatment and the application of cocktails of antiretroviral agents is now the standard of care for HIV patients [2]. Currently over thirty antiviral therapies have been approved for use in HIV-infected patients [3]. However, HAART still suffers from complications with the emergence of multi-drug resistant virus strains, toxicity, drug-drug interactions, difficult treatment regimens, and inadequate pharmacology (bioavailability and tissue distribution) [4,5,6]. Thus, the prevailing belief is that the addition of new anti-HIV agents to HAART regimens will provide additional clinical benefit with the development of new anti-HIV strategies and therapies.

Pyrimidinediones (PYDs) are highly potent, small molecule inhibitors that have a dual mechanism of action against HIV infection: viral entry inhibition and non-nucleoside reverse transcriptase inhibition (NNRTI) [7]. IQP-0410, as well as other highly potent PYD analogs, have shown sub-nanomolar concentration *in vitro* inhibitory activity as reverse transcriptase inhibitors and nanomolar concentration activity as virus entry inhibitors [7,8]. However, one of the biggest obstacles to the administration of small molecule therapeutic products is bioavailability. For example, in studies with Zidovudine (AZT), the first anti-HIV compound approved for clinical use, the therapeutic effectiveness was significantly limited due to its dose-dependent hematological toxicity, low therapeutic index, and, short biological half-life [9]. Additionally, due to first-pass metabolism, the oral bioavailability of AZT was low and the dosage required to maintain therapeutic levels often resulted in toxic concentrations in the blood and other side effects [10]. Similar to other anti-HIV NNRTI’s, IQP-0410 is lipophilic, has low aqueous solubility, and is subject to an extensive first-pass metabolism, resulting in limited therapeutic effectiveness with oral administration [9]. Therefore, non-oral delivery systems may be a means to effectively deliver such lipophilic drugs into the blood plasma and enhance pharmacokinetics [11].

To overcome the described problems associated with conventional therapeutic drug delivery (oral and injectable), controlled drug delivery through formulation is a technology generating significant interest for its ability to enhance the effective drug activity of an active pharmaceutical ingredient (API) through the sustained biomechanical delivery of the API at a controlled rate over time [12]. With conventional dosage forms, the release rate of a drug results in a “peak and trough” profile, where immediately following dosing there is a sharp increase in plasma drug concentration followed by a rapid drop to trough concentrations, which often may fall below effective therapeutic concentration levels. Long term systemic exposure to a drug at modest concentrations is believed to be more beneficial than a bolus supply of drug at higher concentrations [13]. The need to minimize drug concentration fluctuation has led to the development of controlled release drug delivery systems. It has been observed that the benefits of intravenous delivery can be duplicated by using the skin as a portal for drug administration, providing continuous drug infusion into the systemic circulation [14]. Therefore, transdermal drug delivery systems are emerging as an effective method of administering therapeutic products, including anti-HIV agents. Transdermal drug delivery generally refers to the topical application of agents to healthy intact skin either for localized treatment of tissues underlying the skin or for delivery to the systemic circulation. For transdermal products, the goal of dosage design is to maximize the flux of drug product through the skin into the systemic circulation and simultaneously minimize the retention and metabolism of the drug in the skin [15]. Among the various types of transdermal drug delivery systems available for various ailments, including matrix, micro-reservoir, adhesive, and membrane-matrix hybrid, the most common formulation is the incorporation of the drug into the polymer matrix of the transdermal film [16,17]. Transdermal drug delivery systems have several advantages over conventional delivery, such as improved patient compliance during long-term therapy of chronic conditions, reduced undesired side-effects by avoidance of first-pass metabolism and bolus high drug concentrations, sustained drug delivery, maintenance of constant and prolonged drug concentrations in plasma, reduced inter-patient and intra-patient variability, and the opportunity to interrupt or terminate treatment when necessary [18,19,20,21].

Though IQP-0410 has been shown to be a highly potent agent for the therapy of HIV-1 infection, pharmacokinetic studies indicate that IQP-0410 will be subjected to extensive first pass metabolism by the liver. This limits the effectiveness of IQP-0410 when delivered through conventional methods (intravenous, oral, and subcutaneous). The purpose of this study was to prepare a transdermal film containing IQP-0410 and investigate the physicochemical characteristics, *in vitro* release profiles, and *ex vivo* transdermal permeation of IQP-0410 from these films, as well as to assess the efficacy and toxicity of IQP-0410 when delivered from a transdermal film.

## Materials and Methods

### Pyrimidinedione IQP-0410

The pyrimidinedione IQP-0410 was synthesized and provided by Samjin Pharmaceutical Co. LTD (Seoul, Korea) as a dry white crystalline powder. For *in vitro* antiviral and toxicity evaluations, the compound was solubilized in DMSO.

### Virus and Cells

The HIV-1_IIIB_ [22], HIV-1 _BaL_and the CEM-SS cell line [23] were obtained from the NIAID AIDS Research and Reference Reagent Program (Rockville, MD). Peripheral blood mononuclear cells (PBMCs) were isolated from whole blood (Biospecialty Corporation, Colmar, PA) and were phytohemagglutinin (PHA)-stimulated for 72 hours prior to culture in the presence of IL-2.

### Transdermal film formulation

The transdermal films were formulated through a solvent evaporation method. As shown in Table 1, the various film formulations that were initially developed were composed of Ethyl cellulose (EC), Hydroproyl methylcellulose (HPMC), Di-*n*-butyl phthalate, and Propylene glycol. A target dose of 2% (w/w dry mass) per film was defined based upon previously developed PYD formulations with similar activity against HIV-1 [24]. The excipients and IQP-0410 were dissolved in a casting solvent solution of methylene chloride/methanol (75:25, v/v) and combined under continuous mixing from a motorized IKA impeller homogenizer (Wilmington, NC) for 60 minutes at 350 rpm. The homogenized viscous mixture was poured through an Elcometer 4500 film applicator (Rochester Hills, MI) at defined thicknesses (Table 2) to create a thin polymer film. The remaining solvent in the polymer film was evaporated on the film applicator at 37°C for 3 hours to form a solid film sheet. The film sheet was removed from the film applicator, die-cut, and then packaged for storage.

**Table 1:** Formulation of IQP-0410 Dermal Films.

**Formulation**	**EC (%**)	**HPMC (%**)	**PG (%**)	**DnBP (%**)
A	44.0	36.0	8.0	12.0
B	52.0	28.0	8.0	12.0
C	56.0	24.0	8.0	12.0
D	60.0	20.0	8.0	12.0
E	64.0	16.0	8.0	12.0
F	68.0	12.0	8.0	12.0
G	76.0	14.0	8.0	12.0

% *w/w* weight of excipient to total dry weight of film. *EC* ethyl celulose, *HPMC* hydroxypropyl methylcellulose, *PG* propylene glycol, *DnBP* Di-n-butyl phthalate.

**Table 2:** Dermal Film Physical Properties.

**Formulation**	**Thickness (µm**)	**IQP-0410 (%, w/w**)	**IQP-0410 Amount (µg/cm^2^**)	**Appearance**	***InMano* evaluation**
A1	50.0	2.0	294 ± 62.3	Translucent yellow	Smooth; Very low tensile strength; Very high pliability
A2	100	2.0	388 ± 18.9	Translucent yellow	Smooth; Very low tensile strength; Very high pliability
A3	150	2.0	481 ± 31.0	Translucent yellow	Smooth; Low tensile strength; Very high pliability
A4	250	2.0	940 ± 94.4	Translucent yellow	Smooth; Low tensile strength; High pliability
B1	50.0	2.0	294 ± 23.4	Translucent yellow	Smooth; Very low tensile strength; Very high pliability
B2	100	2.0	386 ± 17.9	Translucent yellow	Smooth; Low tensile strength; Very high pliability
B3	150	2.0	449 ± 62.1	Translucent yellow	Smooth; Low tensile strength; Very high pliability
B4	250	2.0	879 ± 87.4	Translucent yellow	Smooth; Low tensile strength; Moderate pliability
C1	250	2.0	907 ± 0.00	Translucent yellow	Smooth; Low tensile strength; Moderate pliability
C2	400	2.0	1105 ± 16.8	Opaque yellow	Smooth; Moderate tensile strength; Moderate pliability
D1	50.0	2.0	315 ± 6.57	Translucent yellow	Smooth; Very low tensile strength; Very high pliability
D2	100	2.0	454 ± 14.0	Translucent yellow	Smooth; Low tensile strength; Very high pliability
D3	150	2.0	448 ± 22.1	Translucent yellow	Smooth; Moderate tensile strength; Very high pliability
D4	250	2.0	668 ± 54.9	Translucent yellow	Smooth; Moderate tensile strength; Moderate pliability
E1	250	2.0	918 ± 98.8	Translucent yellow	Smooth; Moderate tensile strength; Moderate pliability
E2	400	2.0	1168 ± 53.3	Opaque yellow	Smooth; High tensile strength; Low pliability
F1	50.0	2.0	302 ± 77.3	Opaque white	Rough; Very low tensile strength; High pliability
F2	100	2.0	422 ± 31.0	Opaque white	Rough; Low tensile strength; High pliability
F3	150	2.0	453 ± 24.4	Opaque white	Rough; Low tensile strength; High pliability
F4	250	2.0	824 ± 39.4	Opaque white	Rough; Moderate tensile strength; Moderate pliability
G1	100	2.0	214 ± 50.10	Opaque white	Rough; Low tensile strength; Moderate pliability
G2	250	2.0	661 ± 70.8	Opaque white	Rough; Moderate tensile strength; Moderate pliability
G3	400	2.0	1225 ± 30.7	Opaque white	Rough; High tensile strength; Low pliability

### *In mano* transdermal film evaluations

The film formulations were qualitatively evaluated on their general physical characteristics including texture, tensile strength, and pliability for gross acceptability. All qualitative film formulation evaluations were performed *in mano* by a panel of volunteers and then defined from “very low” to “very high” based upon the decision of the panel. For tensile strength evaluations, the films were pulled apart and graded based upon their results. “Very low” tensile strength was defined as the film formulation being unable to maintain any structural integrity when handled. “Low” tensile strength was defined as structural failure with minor *in mano* stress. “Moderate” tensile strength was defined as maintaining structural integrity under stress with the ability to tear the film. “High” tensile strength was defined as a film being unable to be torn. For the film pliability evaluations, pliability was defined as a scale of the formulation’s ability to be rolled and folded. “Low” pliability was defined as a film formulation that could not be rolled nor folded. “Moderate” pliability was defined as a film formulation that could be rolled and when folded produced a permanent crease in the film. “Very high” pliability was defined as a film formulation that was easily rolled and when folded produced no permanent crease.

### Film water content and swelling

The water content and swelling of the transdermal film formulation was measured via the Arizona Instruments VaporPro system (Chandler, AZ). The water content of packaged films cut to 6.45 cm^2^ was evaluated after storage under International Conference of Harmonization (ICH) standard environmental conditions (30°C / 65% Relative Humidity (R.H.)) and accelerated environmental conditions (40°C / 75% R.H.). The hygroscopicity (swelling) of the films under various humidity conditions was evaluated. Films cut to 6.45 cm^2^ were first placed under desiccant conditions for 30 days to obtain a baseline film weight. The dry films were then placed under ambient, 75%, and 90% relative humidity conditions for 30 days. The swelling was calculated as the difference between the swollen film weight and dry film weight with respect to the dry film weight.

### HPLC analytical method

IQP-0410 was quantified by a developed HPLC-UV Vis gradient method through the Shimadzu HPLC system (Columbia, MD). Separation of IQP-0410 was achieved using a HPLC ODS C18 (150 x 4.6 mm, 5 µm) column at 25°C with a mobile phase A (DI water with 0.1% TFA) and a mobile phase B (acetonitrile (ACN)) for 15 minutes at a flow rate of 1.0 mL/min with a lower limit of quantification (LLOQ) of 0.1 µg. The analyte peak was monitored at 267 nm and the retention time of IQP-0410 was determined to be 6.3 minutes.

### *In vitro* dissolution of the transdermal films

The *in vitro* dissolution of the transdermal films and release of IQP-0410 were conducted in a Sotax USP 4 flow-through cell dissolution system (Westborough, MA) as previously reported [25,26,27]. Under sink conditions (100 mL) of dissolution media (10:90 ethanol/DI water), the transdermal films were placed into the dissolution vessel with a flow rate of 10 mL/min at 37°C until the dermal films displayed no additional release of IQP-0410 into the system. The concentration of IQP-0410 released from the films was continuously measured every hour via a Thermo Scientific Evolution 300 UV-Vis spectrometer (Waltham, MA) at 267 nm.

### Film uniformity of IQP-0410

The uniformity of IQP-0410 loading within the film was determined by subdividing the films into fourths. The subdivided film pieces were dissolved in casting solvent solution to rapidly extract the IQP-0410 from the solid films. The extracted solution was diluted with PBS and then analyzed via HPLC analysis.

### *In vitro* release and *ex vivo* permeability of transdermal IQP-0410 films

*In vitro* permeability studies were carried out in Franz cell diffusion cells (PermeGear; Hellertown, PA) via previously described methods [28]. IQP-0410 release rate was evaluated through a 0.45 micron hydrophilic PVDF membrane (Millipore Durapore). The transdermal IQP-0410 films were cut to the area of the Franz Cell chamber (1.54 cm^2^) and placed flush atop the membrane in the donor cell. The receptor cell was filled with a 1:1 Isopropanol (IPA)/Phosphate Buffered Saline (PBS) solution while the donor cell was wetted with 0.1 mL of PBS to maintain humidity [29]. The entire assembly was magnetically stirred and maintained at a temperature of 37°C / 5% CO_2_ for 72 hours. At regular intervals, samples from the receptor compartment of the Franz cell was taken to detect the concentration of IQP-0410 transport through the membrane and analyzed via HPLC.

The *ex vivo* permeability of the IQP-0410 transdermal film was evaluated through full thickness human epidermal tissue (Epiderm FT, MatTek, Ashland, MA). The EpiDerm FT is a tissue model that is comprised of normal, human-derived epidermal keratinocytes (NHEK) which have been cultured to form a multilayered, highly differential model of the human epidermis [30]. The transdermal IQP-0410 films were cut to the area of the Franz Cell chamber (1.54 cm^2^) and placed flush with the apical surface of the epidermal tissue and kept dry. The tissues were maintained in DMEM (MatTek) from the receptor (basal) compartment. The entire assembly was magnetically stirred and maintained at a temperature of 37°C / 5% CO_2_ for 72 hours. At regular intervals, samples from the receptor compartment of the Franz cell were taken and analyzed via HPLC to determine concentration of IQP-0410 that permeated through the tissue into the basal media.

### *In vitro* efficacy of the transdermal films

From the *ex vivo* permeability assays, the IQP-0410 measured in the basal media from the *ex vivo* permeability assay was collected for *in vitro* efficacy evaluations. First, a highly standardized microtiter anti-HIV cytopathic effect (CPE) inhibition assay was performed as previously described [31]. Briefly, the IQP-0410 collected from the permeability assay was serially diluted and added to a 96-well round bottom microtiter plate in triplicate. CEM-SS cells at a concentration of 2.5 x 10^3^ cells per well and HIV-1_IIIB_ at the appropriate pre-determined titer to achieve 90% cell killing at day 6 were sequentially added to the microtiter plate. The cultures were incubated at 5% CO_2_/37°C for six days. Following the incubation, the microtiter plates were stained with XTT (2,3-bis-(2-methoxy-4-nitro-5-sulfophenyl)-2H-tetrazolium-5-carboxanilide) to evaluate the efficacy and toxicity of the test compound(s). AZT was evaluated in parallel as an assay control compound. Secondly, anti-HIV efficacy and cellular toxicity was evaluated in a PBMC-based assay as previously described [32]. Briefly, PHA-stimulated PBMCs cultured in the presence of IL-2 were suspended at 1 x 10^6^ cells/mL and were added to a 96-well round-bottom plate. Serially diluted IQP-0410 was added to the plate in triplicate followed by the appropriate pre-titered strain of HIV-1_BaL_. The culture was incubated for 7 days at 37°C/5% CO_2_. Following the incubation, supernatants were collected for analysis of virus replication by supernatant RT activity and toxicity was assessed by analyzing cell viability utilizing XTT dye reduction. AZT was used as an internal assay control.

### Toxicity of the transdermal films to epidermal tissue

MTT staining (3-(4,5-Dimethylthiazol-2-yl)-2,5-diphenyltetrazolium bromide) was performed on the *ex vivo* epidermal tissues to evaluate toxicity of the transdermal film system. Following the supplied protocol from MatTek, MTT assays were performed on the tissue after film application. Briefly, the transdermal films were applied to the epidermal tissue for either 24 or 72 hours. Following application, the films were removed and MTT solution was added to the tissue and incubated for 24 hours. The decanted solution was read using a Molecular Devices SpectraMax Plus 384 spectrophotometer (Sunnyvale, CA) at 570 nm. The viability of the tissue was calculated as the optical density of the sample after exposure compared to the optical density of the negative control. From the dose response curve, the time at which 50% of the tissue is still viable (ET_50_) was calculated.

### Stability of the transdermal films

A 3-month short term stability protocol of the film formulation was conducted under International Conference on Harmonization (ICH) recommended environmental conditions. Films were packaged in air tight foil pouches and stored under standard conditions (30°C / 65% R.H.) and accelerated conditions (40°C / 75% R.H.). At various time points (0 day, 7 days, 14 days, 1 month, 2 months and 3 months), the films were removed from the chambers and assessed for their water content, IQP-0410 release and stability, content uniformity, and permeability. All tests were performed in triplicate (n = 3 ± SD).

## Results

### Transdermal film formulation

Seven polymeric film formulations (Table 1) were evaluated for potential IQP-0410 transdermal delivery. Because the film applicator allows for direct control of the thickness of the film, various thicknesses and the resulting effect on the qualitative physical characteristics of each film formulation were investigated. The resulting qualitative physical characteristic evaluation of the film formulations are detailed in Table 2. Independent of formulation, as the thickness of the films increased, the amount of IQP-0410 per area of film also increased. The visual appearance of the films generally resulted in a translucent film with only the thickest films or those containing the most ethyl cellulose (series “F” and “G”) producing an opaque film. The film formulations were all smooth *in mano* with the exception of formulation series “F” and “G”. Film formulations that were defined as “very low” and “low” tensile strength were removed from further development. Additionally, film formulations that were defined as “low” to “moderate” pliability were similarly removed from development. Therefore, from the matrix of film formulations produced, film formulation D was identified as satisfying the most qualitative characteristics containing 60% (w/w) EC, 20% HPMC, 8% Propylene glycol, and 12% Di-*n*-butyl phthalate. Specifically, the formulation subset D3, with a film thickness of 150 µm, was identified as the lead formulation for further development. The lead film formulation D3 at 2% (w/w) drug loading resulted in a dosing concentration of 448 ± 22.1 µg/cm^2^ (Table 2).

### Water Content and Film swelling

**Figure 1:**
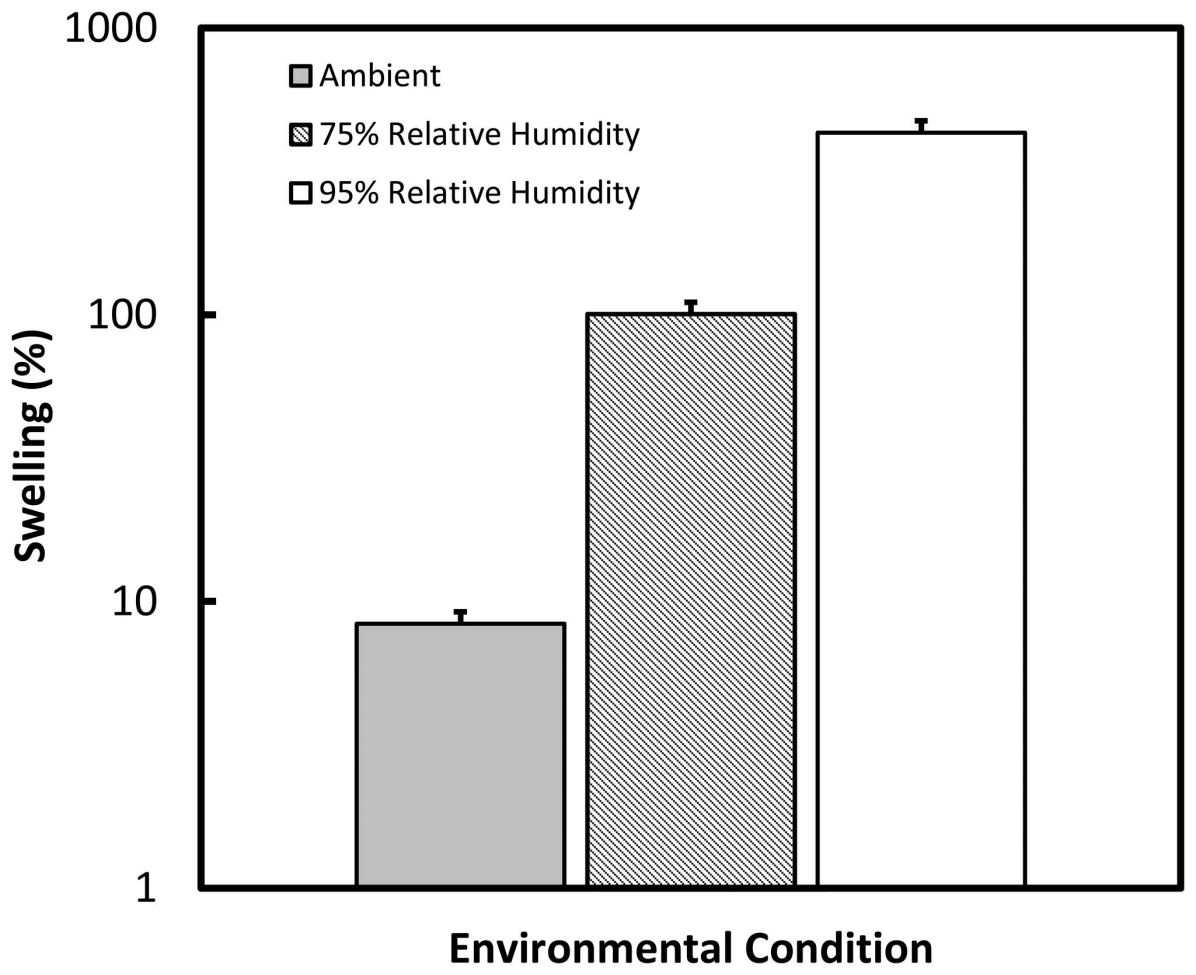
Swelling of the transdermal patches. The patches were placed under various environmental conditions: ambient humidity (grey), 75% Relative humidity (striped), and 95% relative humidity (white). The patches remained under these conditions until a stable mass was measured. Swelling was calculated from the percent change in film weight from water uptake. n = 3 ± standard deviation (SD).

The water content of the films was monitored as part of the stability protocol for 3 months under standard and accelerated environmental storage conditions. The initial water content of the films was recorded as T = 0 and are reported as a percentage of water in the film (Table 3). The films immediately after manufacturing contained 1.15 ± 0.26% (w/w) water. Over three months, under both environmental storage conditions, the films maintained their water content within acceptable variation ranges with no significant gain of water, indicating proper packaging. The swelling of the films was performed under ambient, 75% R.H. and 95% R.H. environmental conditions (Figure 1). After storing the films under each humidity condition until the weight of the films stabilized, the films displayed hygroscopic behavior as a +430 ± 31.3% swelling was measured when the films were placed in a 95% relative humid environment. When left under ambient conditions, the transdermal films showed a swelling of +8.35 ± 0.41%.

**Table 3:** Film Water Content

**Formulation D3**	**Water Present (% w/w**)	
	**30°C / 65%R.H.**	**40°C / 75%R.H.**
0 days	1.15 ± 0.26	
7 days	1.48 ± 0.60	1.28 ± 0.42
1 month	1.22 ± 0.23	0.56 ± 0.05
2 months	1.30 ± 0.00	1.13 ± 0.48
3 months	0.79 ± 0.18	0.94 ± 0.08

### *In vitro* film dissolution and IQP-0410 content uniformity

**Figure 2:**
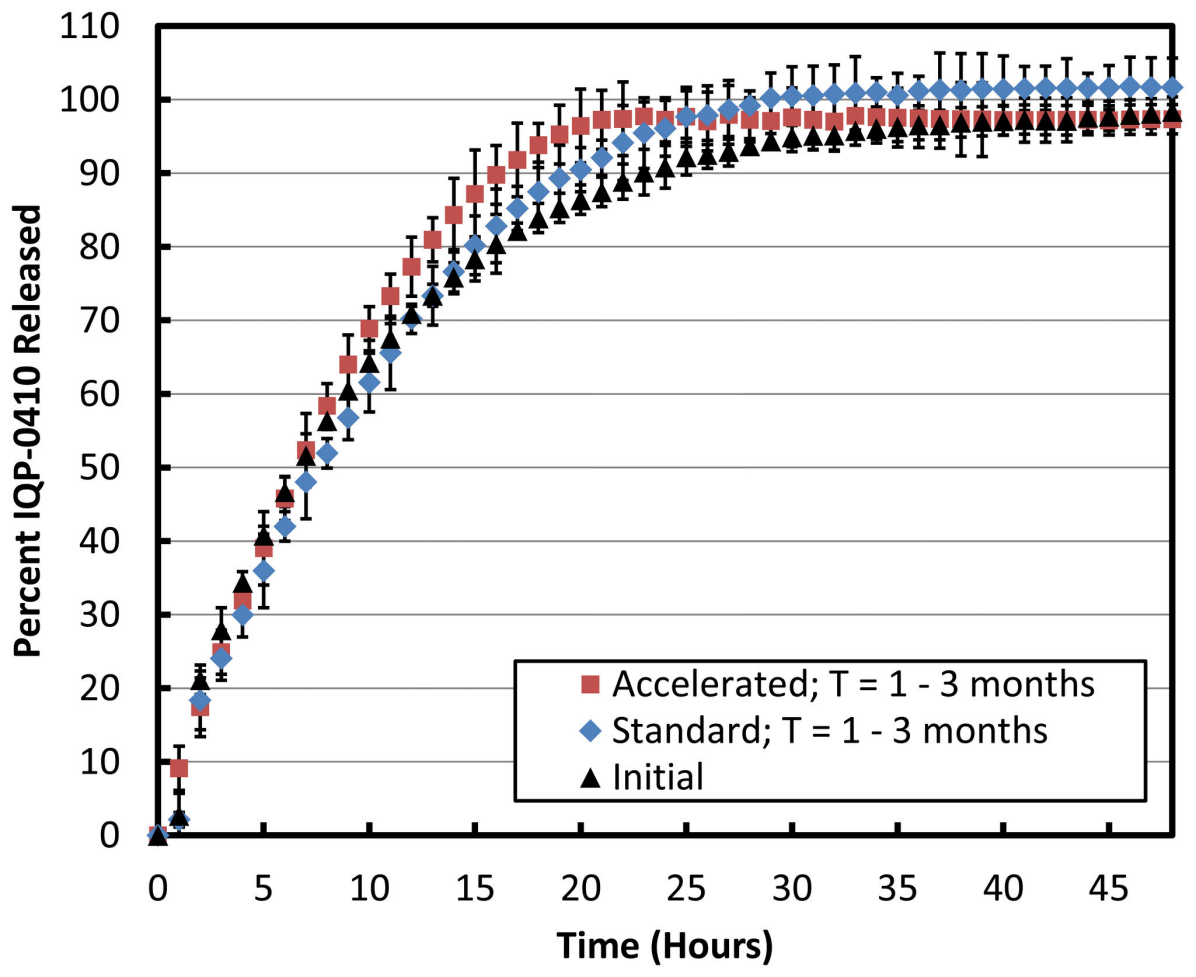
*In vitro* release for IQP-0410 from transdermal films. Under 10:90 EtOH-Water sink conditions, the *in* *vitro* release of IQP-0410 from the transdermal films were evaluated in a USP-4 apparatus for 48 hours upon initial manufacturing (black triangles) (n = 3 ± SD). The transdermal films were packaged into air-tight foil pouches and stored under standard (30°C/65% R.H.) and accelerated (40°C/75% R.H.) environmental conditions for 3 months. At 1 month, 2 months, and 3 months, the transdermal films were removed from storage and tested for drug release. The combined average (n = 9 ± SD) of the transdermal films stored under standard environmental conditions for 3 months (blue diamonds). The combined average (n = 9 ± SD) of the transdermal films stored under accelerated environmental conditions for 3 months (red squares).

The *in vitro* dissolution of the transdermal films was performed to evaluate the release and recovery of IQP-0410 from the film formulation. Under both standard and accelerated storage conditions over three months, the transdermal films resulted in a near complete recovery of IQP-0410 after 26 hours in the 10:90 ethanol/water dissolution media (Figure 2, Table 4). When subdivided, the transdermal film showed a high IQP-0410 distribution uniformity in all films stored at standard and accelerated storage conditions for 3 months with an overall root mean-squared deviation (RSD) of ≤ 5.3% (Table 4).

**Table 4:** Drug Stability of IQP-0410 Dermal Films.

**Formulation D3**	**30°C / 65%R.H.**		**40°C / 75%R.H.**	
	**IQP-0410 Recovery**	**Film Uniformity (% RSD**)	**IQP-0410 Recovery**	**Film Uniformity (% RSD**)
0 days	100 ± 0.60%	1.02%	100 ± 0.60%	1.02%
7 days	93.6 ± 1.36%	1.46%	97.7 ± 3.34%	3.42%
1 month	99.5 ± 2.01%	2.90%	102 ± 4.78%	2.46%
2 months	123 ± 2.45%	1.99%	123 ± 9.29%	5.29%
3 months	96.5 ± 4.94%	5.12%	102 ± 7.27%	4.27%

### *In vitro* release and *ex vivo* permeability of transdermal IQP-0410 films

**Figure 3:**
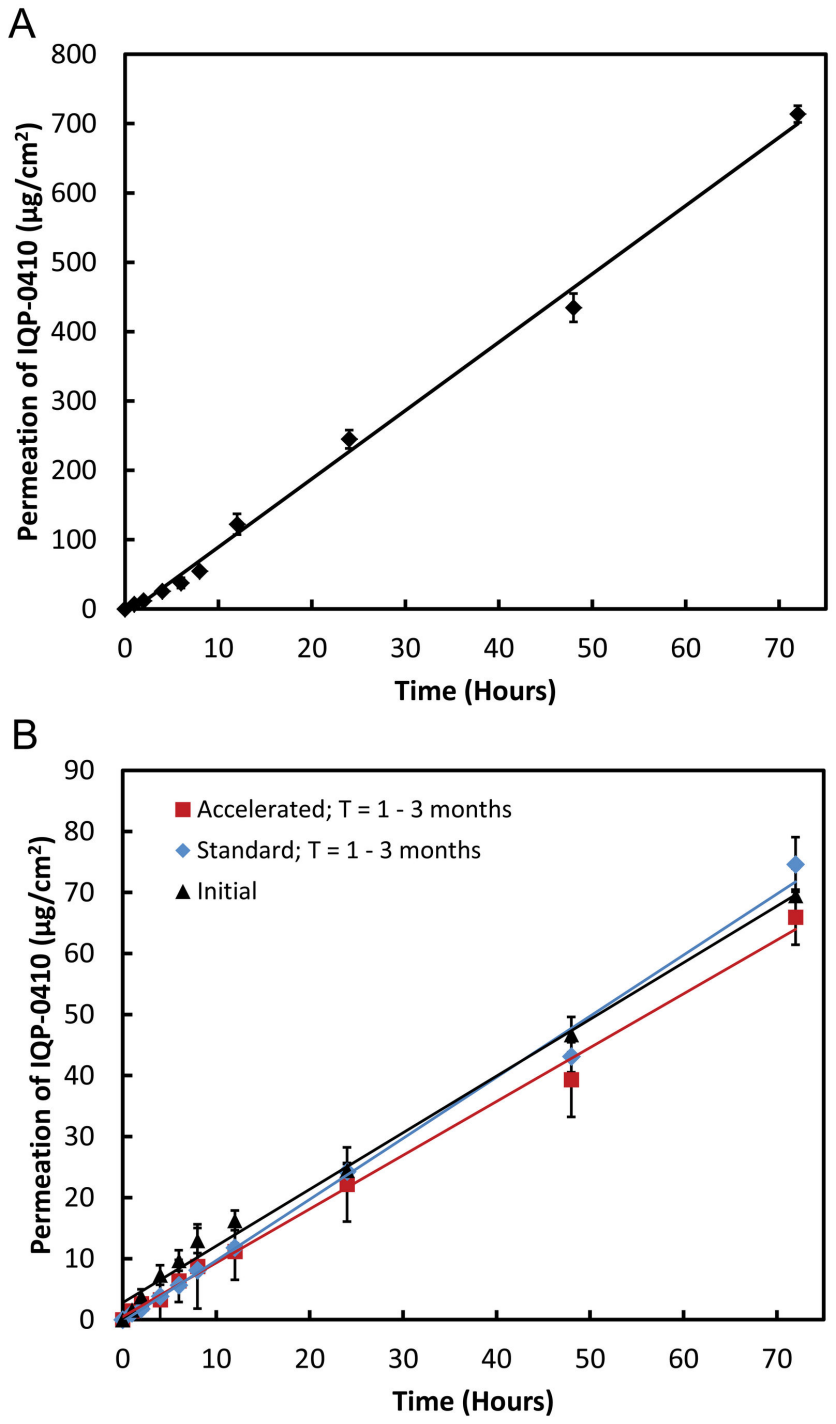
*In vitro / ex vivo* permeability of the IQP-0410 transdermal film through barrier. The *in* *vitro* release and subsequent transport of IQP-0410 from the transdermal film through a PVDF membrane over 72 hours (A) (n = 3 ± SD). The *ex* *vivo* release and permeability of IQP-0410 from the transdermal films through full thickness epidermal tissue over 72 hours (B – *black* *triangles*) (n = 3 ± SD). The transdermal films were packaged into air-tight foil pouches and stored under standard (30°C/65% R.H.) and accelerated (40°C/75% R.H.) environmental conditions for 3 months. At 1 month, 2 months, and 3 months, the transdermal films were removed from storage and tested for drug release and permeability through full thickness epidermal tissue. The combined average (n = 6 ± SD) of the transdermal films stored under standard environmental conditions for 3 months (B - *blue* *diamonds*). The combined average (n = 6 ± SD) of the transdermal films stored under accelerated environmental conditions for 3 months (B - *red* *squares*).

The release of IQP-0410 from the transdermal film and transport through the PVDF membrane resulted in a linear accumulation of IQP-0410 in the receptor cell (Figure 3A). From the slope of the line, the overall drug flux of IQP-0410 through the PVDF membrane was calculated as 9.84 µg/cm^2^/hr. From this transport rate, the 1.54 cm^2^ film is calculated to have a potential complete release of IQP-0410 from the film through the membrane in 45 hours. In the *ex vivo* permeability evaluation, the IQP-0410 transdermal films were applied to full thickness epidermal tissue for 72 hours (Figure 3B). The transdermal film produced a linear drug release and permeability through the skin tissue over the 72 hour application. In comparison to the *in vitro* synthetic membrane transport assay, the *ex vivo* permeability resulted in a 10-fold reduction in drug flux of 0.94 ± 0.06 µg/cm^2^/hr.

### Toxicity of the IQP-0410 transdermal films to skin

The toxicity of the IQP-0410 transdermal film to epidermal tissue was evaluated via MTT endpoint analysis following a 24 hour and a 72 hour exposure. Following the 24 hour exposure, the epidermal tissue viability was measured at 121 ± 19.8% of control tissue. Following 72 hours of exposure, the tissue viability was 91.2 ± 9.71% of control tissue. Triton-X was used as an assay control and had a viability of 46%.

### *In vitro* anti-HIV activity of released IQP-0410

The concentrations of IQP-0410 that were released from the film and permeated through the epidermal tissue into the basal media were collected and measured via HPLC on days 1 (45.9 ± 20.8 µM), 2 (94.5 ± 12.7µM), and 3 (134 ± 14.7 µM) of film application. The efficacy of the collected IQP-0410 released from the films through the epidermal tissue was evaluated in HIV-1 efficacy assays utilizing CEM-SS cells to evaluate the inhibition of HIV-1-induced cytopathic effects and PBMCs to evaluate inhibition of virus replication (Table 5). The basal media collected IQP-0410 resulted in no *in vitro* toxicity to CEM-SS cells and or PBMCs at the highest drug concentrations evaluated (> 15 µM). After 3 days of application, IQP-0410 that permeated through the tissue and collected in the basal media resulted in an *in vitro* EC_50_ value of 1.71 ± 0.01 nM to 2.56 ± 0.40 nM in CEM-SS cells and 0.58 ± 0.03 nM to 1.65 ± 0.55 nM in PBMC’s.

**Table 5:** *In Vitro* Efficacy of IQP-0410 Dermal Films.

**Time (days**)	**Antiviral Efficacy**	**Antiviral Efficacy**
	**CEM-SS/HIV-1_IIIB_**	**PBMC/HIV-1_BaL_**
	**EC_50_ (nM**)	**EC_50_ (nM**)
1	1.71 ± 0.01	1.65 ± 0.55
2	1.99 ± 0.16	0.82 ± 0.26
3	2.56 ± 0.40	0.58 ± 0.03

### Stability of the transdermal films

The stability of IQP-0410 transdermal films packaged in foil pouches was evaluated for 3 months under standard environmental conditions (30°C / 65% R.H.) and accelerated environmental conditions (40°C / 75% R.H.). Upon evaluation of film moisture content (Table 3), IQP-0410 assay (Table 4), film dissolution and IQP-0410 release (Figure 2), and IQP-0410 permeability (Figure 3), the transdermal film formulation resulted in no significant deviations from target formulation values.

## Discussion

The pyrimidinedione IQP-0410 has been identified as a potent antiretroviral therapeutic for HIV treatment. In developing the NNRTI as a potential therapeutic product, transdermal films were investigated as a formulation to systematically deliver IQP-0410 over extended periods of time to avoid bolus administration and first-pass metabolism. However, in considering transdermal drug delivery, the barrier properties of epidermal tissue must be considered. Transport through the epidermis is primarily diffusion driven, governed by the physicochemical properties of the API and the barrier itself [33]. The drug released from the patches diffuses through the boundary layer (stratum corneum) to the underlying dermal layer and into the blood vessels. The rate and ultimate success of a drug to diffuse through the skin is largely dependent upon its molecular weight, partition coefficient (Log P), solubility, and polarity. IQP-0410 is practically insoluble and has a molecular weight of 352.43 g/mol. With a calculated Log P of 3-4, IQP-0410 is non-polar and thus lipophilic. Compounds with Log P values of 2-3 show optimal permeability across the stratum corneum as well as moderate partitioning out of the stratum corneum. However, compounds with Log P values > 3 show a high diffusion into the stratum corneum with little transport into the systemic circulation [34]. The lipophilicty of IQP-0410 may result in increased residence times in the stratum corneum, limiting systemic delivery. *In vitro* studies show that IQP-0410 is efficacious at sub-nanomolar concentrations against HIV-1 (0.28 nM) with a resulting therapeutic index of greater than 500,000 [8]. However, in *vivo* PK and bioavailability studies in mice have shown that IQP-0410 only has a 24% oral bioavailability with a half-life of 5.37 hours and an intravenous half-life of 30 minutes. This short systemic residence time can be attributed by extensive first-pass metabolism by the liver. Therefore, by-passing oral first-pass metabolism via dermal delivery may not be an issue with IQP-0410 having limited diffusion from the stratum corneum into the underlying circulation. In this study, the NNRTI IQP-0410 was formulated into a transdermal film formulation and evaluated for potential ARV drug delivery.

From seven initial film formulations, a matrix of transdermal films was produced with varying product thicknesses. The film thickness had a direct effect on the amount of IQP-0410 loaded into a film. As thickness increased, the amount of loaded IQP-0410 per area increased at a ratio of ≥ 5:3. The only exception was formulation series “D” which had more stable 5:2 ratio of increased thickness to loaded IQP-0410. An initial lead formulation was identified through qualitative *in mano* evaluations that defined appearance, *in mano* tensile strength, and pliability. Formulations were removed from consideration if they were defined by the panel as having “very low” to “low” *in mano* tensile strength, as these formulations could not be handled, or if their pliability was defined as “low” to “moderate”, as these formulations would not allow for any flexibility when applied. Therefore, formulation series D, specifically sub-formulation “D3” was identified as the lead film formulation for development in this study. The resulting film was a smooth translucent film that can easily conform to the contours of the arm with a thickness of 150 µm and a drug loading of 448 ± 22.1 µg/cm^2^.

The transdermal films were manufactured to have a water content of 1-5% (w/w) to produce a stable polymer film matrix but still allow for enough pliability to avoid issues with the films being dry and brittle. The film formulation under development had a water content of 1.51 ± 0.26% which corresponds to 0.19 µL/cm^2^ of water. Overall, the films showed significant swelling when exposed to high levels of humidity. At a 95% relative humidity environment, the films resulted in swelling of 430% from a completely dried film. However, under ambient conditions, the films only resulted in a swelling of 8.35%. The primary excipient in the films is ethyl cellulose, a hydrophobic polymer, which will limit film hygroscopy and swelling. However, the inclusion of HPMC, a hydrophilic polymer, is responsible for resulting in a film that is water-permeable and subject to swelling [35]. This hydration loosens the polymer matrix which then allows for the drug to be released from the film. When sealed into packaging, the film resulted in no increase of water content when stored at 30°C / 65% R.H. and 40°C / 75% R.H. for up to 3 months.

In the dissolution media, the cumulative amount of IQP-0410 recovered from the film formulation was near 100%. In films immediately tested and films tested over 3 months that were stored at standard and accelerated conditions, all films resulted in complete IQP-0410 release and recovery after 26 hours. The rapid release of IQP-0410 from the films in the dissolution media could be explained by the hydrophobic nature of the ethyl cellulose. While ethyl cellulose limits film hygroscopy, it readily solubilizes in non-aqueous solutions such as ethanol. Therefore, with a dissolution media containing both ethanol and water, the entire film is rapidly swelling to allow for a rapid release of IQP-0410. Another reason for the rapid *in vitro* release is the inclusion of Di-*n*-butyl phthalate, which has been demonstrated to enhance *in vitro* release [36]. There was observed a minor increase in the release rate of IQP-0410 from the films stored under accelerated conditions. While not significant, it is was observed that these films *in mano* were more pliable that the films stored at standard conditions. The increased pliability due to the heat may reduce the integrity of the film polymer matrix and may contribute to the slightly faster release of IQP-0410 into dissolution media measured; however, the cumulative recovered IQP-0410 was unaffected. This rapid release rate, however, shouldn’t be indicative of the actual release of IQP-0410 from the transdermal film when applied to a barrier as optimally there will be little media when the films are applied to cause premature drug release. Regardless, these *in vitro* release studies demonstrate that formulation of IQP-0410 into the polymeric transdermal films does not negatively affect API recovery. Additionally, the films manufactured showed a uniform distribution of IQP-0410 through the film with an RSD of < 5.29% overall.

The *in vitro* / *ex vivo* release and permeability studies of IQP-0410 from the transdermal films were performed on synthetic PVDF membranes and epidermal tissues, respectively. When applied to the membrane and moistened, the transdermal films displayed a linear release of IQP-0410 across the membrane into 1:1 IPA/PBS solution. While the flux of IQP-0410 across the membrane is not a true measurement of drug delivery and permeability, the drug transport of IQP-0410 from the transdermal film across the membrane does correspond to a zero-order release kinetic profile [33]. Therefore, with a calculated flux of 9.83 µg/cm^2^/hr, we calculate a potential complete release of IQP-0410 through the membrane in 1.75 days.

When applied to epidermal tissue for 3 days, the transdermal films resulted in a linear zero-order release rate through the tissue into the basal media. While similar transport behavior was observed in the membrane *in vitro*, the flux of IQP-0410 through the *ex vivo* epidermal tissue was calculated to be 10-fold slower at 0.94 ± 0.06 µg/cm^2^/hr. As such, the predicted complete release of IQP-0410 from the film is calculated to occur after 19.5 days from application and 50% release is calculated to occur after 9.75 days of use. The reduction in drug flux is to be expected and can be directly attributed to the difference in diffusion of IQP-0410 through epidermal tissue and a thin membrane. Most importantly, despite initial predictions that a drug with the physicochemical properties of IQP-0410 would remain in the stratum corneum, IQP-0410 was successfully released and permeated through the full thickness epidermal tissue to be collected in the basal media suggesting the potential viability of controlled zero-order delivery of IQP-0410 through the skin.

While the *in vitro* release of IQP-0410 into dissolution media suggested an increased rate from films stored under accelerated storage conditions, this was not observed in the *ex vivo* permeability studies. Transdermal drug delivery has two parts for overall drug delivery: release from formulation to the epidermis, and permeation through the skin to the underlying blood vessels. Therefore any increases in drug release rate from the film formulation will be mitigated by the diffusion of the drug through the tissue. The passive diffusion across the skin, the basis of transdermal drug delivery, will be the limiting factor in the drug delivery and permeability [37]. Only through external interventions to increase skin permeability, such as chemical solubility enhancers, thermal ablation, microneedles, and iontophoresis, will increased drug release from the formulations effect overall transdermal film drug delivery [38].

To evaluate the anti-HIV efficacy of the delivered IQP-0410 through the epidermal tissue, *in vitro* assays in CEM-SS cells and PBMC’s against HIV-1 were performed with the collected IQP-0410 from the basal media. The concentration of IQP-0410 detected in the basal media was defined as the amount of IQP-0410 potentially bioavailable systemically and then was used to evaluate the *in vitro* anti-HIV efficacy and cellular toxicity performed in CEM-SS cells and PBMCs. In the basal media, 45.9 ± 20.8 µM, 94.5 ± 12.7µM, and 134 ± 14.7 µM of IQP-0410 was collected each day, respectively, resulting in an average EC_50_ value of 2.09 ± 0.43 nM in CEM-SS cells and 1.02 ± 0.56 nM in PBMCs over a three day application. Therefore, in conjunction with the drug recovery evaluations, we are assured that this transdermal film formulation has neither negative physicochemical nor biological effects on IQP-0410.

Ultimately it is important to evaluate whether the transdermal film is capable of delivering appropriate amounts of drug into the system. *Ex vivo* release studies performed over three days determined that the concentration of delivered IQP-0410 after 24 hours (46 µM) is 5,000 fold greater than *in vitro* EC_95_ values [8]. It is understood that the *ex vivo* concentrations of permeated IQP-0410 are not completely representative of potential systemic *in vivo* behavior; however, it can be concluded that efficacious concentrations of IQP-0410 are passing through the skin. In addition, previous product profile studies of IQP-0410 have shown that *in vivo* oral administration in mice results in 24% oral bioavailability with a half-life of 5.37 hours and in the presence of liver microsomes, the expected half-life in humans is 15.7 minutes. Therefore, it will be critical to avoid first pass metabolism in the liver commonly observed in oral administration to maintain therapeutic concentrations of IQP-0410 in the blood plasma by controlled delivery through the skin.

One the greatest disadvantage to transdermal delivery is the possibility that a local irritation will develop at the site of administration. Irritation can be caused by the drug itself, the adhesive, or other excipients in the formulation. When applied to the skin tissue for 24 and 72 hours, the IQP-0410 transdermal films had a tissue viability of 121 ± 19.8% after a 24 hour exposure and 91.2 ± 9.71% tissue viability after 72 hours. The *in vitro* toxicity results from the tissue do offer a correlation in predicting clinical *in vivo* skin irritation [30]. So, from the MTT analysis of the IQP-0410 transdermal film, an *in vitro* ET-50 >24 hours is equivalent to the non-irritancy of 10% Tween-20. Therefore, it is expected that the IQP-0410 film formulation will result in no adverse skin irritation when administered.

When packaged in air-tight foil pouches, the transdermal films’ physical properties, *in vitro/ex vivo* IQP-0410 release and permeability, toxicity, and anti-HIV efficacy did not significantly deviate from target values. These results suggest that for 3 months at both standard and accelerated environmental conditions, the formulated IQP-0410 transdermal films are stable products.

## Conclusions

The pyrimidinedione IQP-0410 is a potent NNRTI that has significant potential as an anti-HIV therapeutic agent. Its product profile suggests it will experience many of the absorption, distribution, metabolism, and excretion (ADME) issues observed in other molecules of this class. Therefore, transdermal drug delivery was investigated as a potential dosage form to overcome these issues. A polymeric based transdermal film was formulated to hold and deliver IQP-0410 that was composed of non-toxic excipients. Our *in vitro* and *ex vivo* studies successfully demonstrated that IQP-0410 could be released from the transdermal films and delivered through a full thickness epidermal tissue model. The subsequent successful *in vitro* reduction of HIV-1 activity from the delivered drug over a 3 day application suggests the potential of IQP-0410 to be administered via transdermal patches. Further studies investigating the transdermal delivery of IQP-0410 will potentially result in transdermal patches that would offer an easier option for patients to comply with their medication regimes as compared to current treatments. 
